# How conserved are the conserved 16S-rRNA regions?

**DOI:** 10.7717/peerj.3036

**Published:** 2017-02-28

**Authors:** Marcel Martinez-Porchas, Enrique Villalpando-Canchola, Luis Enrique Ortiz Suarez, Francisco Vargas-Albores

**Affiliations:** 1 Centro de Investigación en Alimentación y Desarrollo, A.C., Hermosillo, Sonora, Mexico; 2 Instituto Tecnológico de Morelia, Morelia, Michoacán, Mexico

**Keywords:** Kmers, Biodiversity, Conserved regions 16S, Primer design

## Abstract

The 16S rRNA gene has been used as master key for studying prokaryotic diversity in almost every environment. Despite the claim of several researchers to have the best universal primers, the reality is that no primer has been demonstrated to be truly universal. This suggests that conserved regions of the gene may not be as conserved as expected. The aim of this study was to evaluate the conservation degree of the so-called conserved regions flanking the hypervariable regions of the 16S rRNA gene. Data contained in SILVA database (release 123) were used for the study. Primers reported as matches of each conserved region were assembled to form contigs; sequences sizing 12 nucleotides (12-mers) were extracted from these contigs and searched into the entire set of SILVA sequences. Frequency analysis shown that extreme regions, 1 and 10, registered the lowest frequencies. 12-mer frequencies revealed segments of contigs that were not as conserved as expected (≤90%). Fragments corresponding to the primer contigs 3, 4, 5b and 6a were recovered from all sequences in SILVA database. Nucleotide frequency analysis in each consensus demonstrated that only a small fraction of these so-called conserved regions is truly conserved in non-redundant sequences. It could be concluded that conserved regions of the 16S rRNA gene exhibit considerable variation that has to be considered when using this gene as biomarker.

## Introduction

The study of microbial communities through sequencing the 16S rRNA gene by Sanger method has been abandoned by most of the scientists. Instead, high throughput sequencing technology has emerged as a masterpiece for the robust study of microbial communities, allowing laboratories to obtain millions of high quality sequences (Martínez-Porchas & Vargas-Albores, 2015; Yang, Wang & Qian, 2016). Whether this is a significant improvement for the discipline, the short size of these sequences is a limiting factor for the taxonomic classification of bacteria and archaea.

A novel technology based on single molecule real-time sequencing (SMRT), has been tested as possible solution for this problem with promising results. However, the per-base sequencing cost is still significantly higher than that of current high throughput sequencing platforms, and thus inviable for most of the laboratories (Schloss et al.,  2016; Singer et al., 2016). Therefore, the use of 16S rRNA fractions to study bacterial diversity seems to be the main strategy during the following years. Furthermore, there are specific studies focused on particular taxonomic groups of bacteria that would require a specific fraction of the 16S rRNA gene (Li et al., 2009; Pfeiffer et al., 2014).

In this regard, several primers targeting diverse hypervariable regions of the 16S rRNA gene have been used and reported as guarantee of wide coverage and good amplification (Hiergeist, Reischl & Gessner, 2016; Takahashi et al., 2014; Yang, Wang & Qian, 2016); however, none of these primers is truly universal and the coverage usually depends upon intrinsic factors such as primer design (sequence, size, position, degenerations, combinations), chemical reagents used, amplification conditions and other PCR biases, and extrinsic factors such as the kinds of samples (bacterial composition and environment) and PCR inhibitors hauled in the sampling process (Albertsen et al., 2015; Tremblay et al., 2015; Walker et al., 2015). Moreover, regions of the 16S rRNA gene differ in taxonomic informativeness (Kumar et al., 2011; Soergel et al., 2012); thus, some regions seem to be more useful for taxonomic classification from a general perspective and others for particular taxa.

Most of the studies regarding proposal and effectiveness of different primers are usually based on the study of biological samples. These studies have been useful to extend the panorama regarding the research of bacterial communities (Cruaud et al., 2014; Logares et al., 2014); however, the intrinsic and extrinsic factors influencing the performance of these primers do not allow concluding if they have the best possible coverage. These kinds of results allow to conclude if one pair of primers is better than others, but do not provide conclusive information regarding coverage; for example, it is possible that only a fraction of the species thriving in any environmental sample are being covered by any combination of primers, whereas the 16S rRNA fraction of others may require different amplification conditions, or do not match with the primer sequence because some fragments of the conserved regions are probably not as conserved as expected, and so on. In this case, the information provided by environmental samples regarding the coverage of any pair of primers would be incomplete.

Furthermore, many of the primers used are frequently not validated through *in silico* tests, while others are proved only with a couple of thousand sequences previously selected (Morales & Holben, 2009; Peabody et al., 2015). Additionally, the use of degenerated primers has been proposed for the amplification of DNA coding for homologous genes, covering a larger number of genes from unspecific prokaryotes. Degenerated primers were initially designed manually, inserting degenerations after multiple alignments; however sophisticated software programs are used today (Linhart & Shamir, 2002; Najafabadi, Torabi & Chamankhah, 2008; Qu et al., 2012). Thus, it is necessary to understand variations in conserved regions of the 16S rRNA gene and to carry out tests with all possible sequences (including degenerations) and combinations; which is impractical and unfeasible. However, this can be done virtually considering all of the information contained in robust databases, not only the sequences obtained from biological samples. Moreover, the analysis of these conserved regions could provide useful information to evaluate how much conserved are these regions and which sequence positions are truly conserved. Therefore, the aim of this study was to evaluate the conservation degree of the so-called conserved regions flanking the hypervariable regions of the 16S rRNA gene.

## Materials and Methods

The conservation degree of the regions flanking all of the nine-hypervariable regions of the 16S rRNA gene was estimated through the analysis of data contained in the high quality ribosomal RNA database SILVA SSU Ref NR 99 (release 123) which have non-redundant bacterial sequences with at least 1,200 bases length.

**Figure 1:**
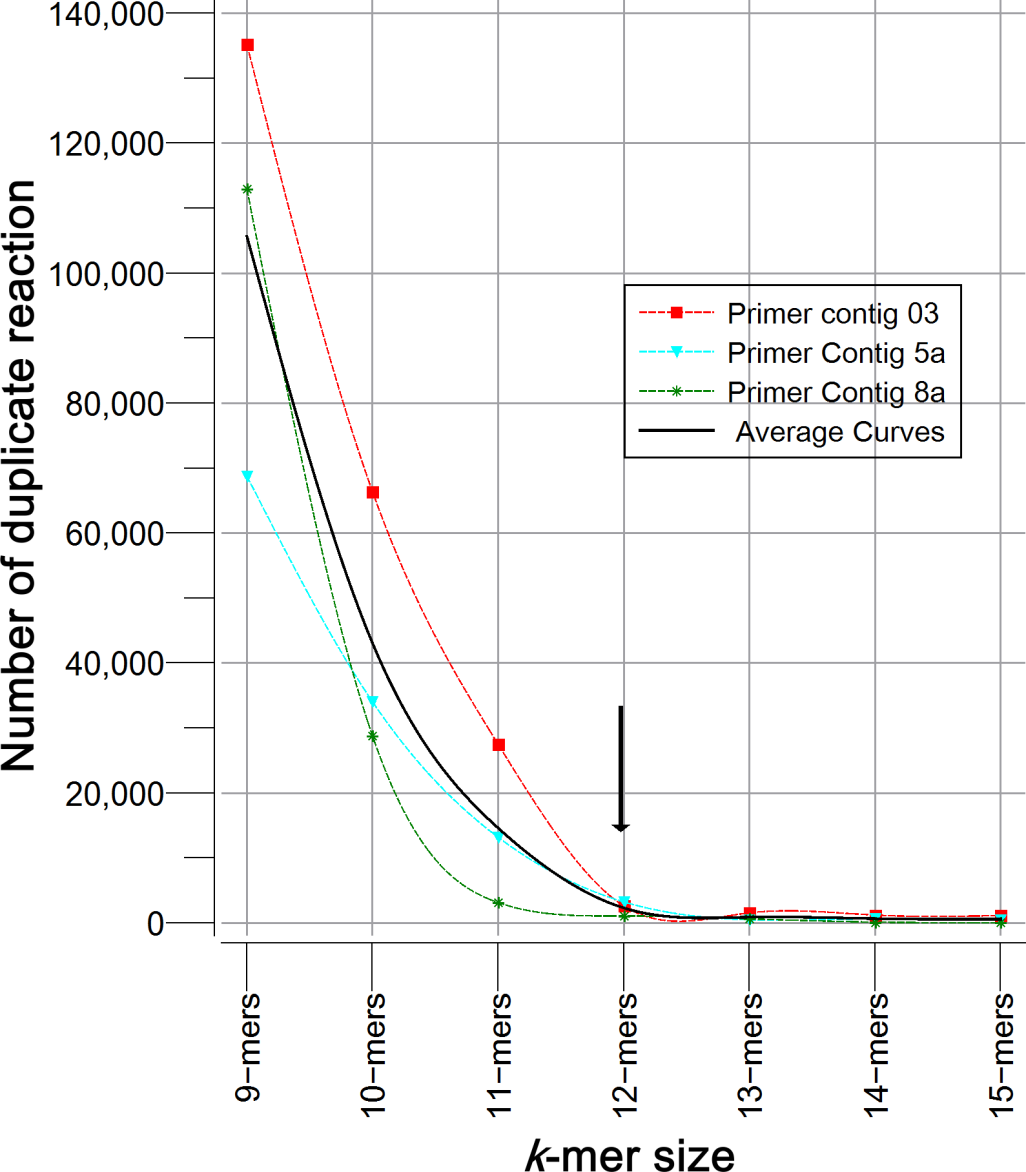
Specificity analysis of *k*-mers with different size (9 to 15 nucleotides). Figure describes the number of sequences of 16S rRNA gene (Silva 123) that showed duplicate reaction. *k*-mers tested corresponded to primer contigs 3, 5a and 8a. As expected, the longer the *k*-mer the greater astringency, and the probability of finding a duplicate is reduced. The optimal size was determined by the inflection point.

Homemade PHP scripts were used for searching specific nucleotide chains, recovering fragments of bacterial sequences, making calculations and ordering information. The following process was carried out: all of the primers reported for each region were aligned to generate a continuous “primer contig” sequence in order to perform a sequence-scan analysis of regions (position by position); if primers formed separated contigs by a gap, each segment was considered as sub-contig (a, b or c). Previous tests using 9- to 15-mers, revealed that greater specificity was achieved with 12- to 15-mers, but a higher proportion of sequences were obtained with 12-mers (Fig. 1). Thereafter, sequences sizing 12 nucleotides (12-mers) were extracted from the primer contigs.

The number of 12-mers to prove was calculated by the following equation: *Number of 12-mers* = *consensus size − k* + *1*; where, *k* was the kmer size (12 in this case). If the 12-mer contained degenerations, each isoform was considered for the analysis; for example, nucleotide ambiguity code establishes that keto or K represents T or G, therefore sequence containing this degeneration was multiplied by two possibilities. This was also considered for all kinds of degenerations detected in all sequences; for instance, Y, M, S, R, W = 2 possibilities, V, H, B, D = 3, and *N* = 4. Thus, the primer contig sequence(s) for each region was broken down into 12-mers and its respective isoforms replacing any degeneration by the corresponding nucleotides. Finally, the exact sequence of each 12-mer isoforms generated from all conservative regions was searched into the entire set of sequences contained in SILVA database.

Equivalent regions of primer contigs 2, 3, 4, 5b, 6a, 7a, 8a and 9 were recovered from all sequences of SILVA database using the 12-mer registering the highest frequency. After the position (full match) with the most frequent 12-mer was detected, nucleotides flanking both extremes of these 12-mers were added as many as necessary to achieve contig size. To avoid induced biases by repeated sequences only those non-redundant (NR) were considered. The frequency of the nucleotides occupying each position was determined in both set of sequences, all and NR. In case of degeneracy, each base received the proportional value. For example, for R (A/G), 0.5 of A and 0.5 of G was considered.

## Results and Discussion

16S rRNA sequence analyses which assume that conserved regions allow for the design of universal primers have been performed to elucidate the taxonomic affinities of a wide range of taxa and to robustly assessing the prokaryotic diversity of environmental samples (Baker, Smith & Cowan, 2003; Martínez-Porchas & Vargas-Albores, 2015; Martinez-Porchas, Villalpando-Canchola & Vargas-Albores, in press; Wang et al., 2016; Wang & Qian, 2009). Fifteen primer contigs with lengths ranging from 16 (primer contig 6b) to 44 nucleotides (primer contig 3) were constructed by assembling all reported primers designed for matching the conservative regions (Table 1).

**Table 1:** Primer contigs generated by assembling all of the primers reported for each conserved region of the 16S rRNA gene. Location is based on *E. coli* sequence.

Name	Sequence	Location	References
1	AGAGTTTGATYMTGGCTCAG	8–27	(Edwards et al., 1989; Isenbarger et al., 2008; Kumar et al., 2005; Ludwig, Mittenhuber & Friedrich, 1993; McInerney et al., 1995; Muyzer et al., 1995; Oguntoyinbo, 2007; Suzuki & Giovannoni, 1996; Wilson, Blitchington & Greene, 1990)
2	ASYGGCGNACGGGTGAGTAA	100–119	(Schmalenberger, Schwieger & Tebbe, 2001; Sundquist et al., 2007)
3	ACTGAGAYACGGYCCARACTCCTACGGRNGGCNGCAGTRRGGAA	320–363	(Amann et al., 1990; Baker, Smith & Cowan, 2003; Chakravorty et al., 2007; Dethlefsen et al., 2008; Fierer et al., 2008; Hang et al., 2014; Hansen et al., 1998; Lane, 1991; Muyzer, De Waal & Uitterlinden, 1993; Reysenbach & Pace, 1995; Rudi et al., 1997; Walter et al., 2000; Wang & Qian, 2009; Watanabe, Kodama & Harayama, 2001; Wuyts et al., 2002)
4	GGCTAACTHCGTGNCVGCNGCYGCGGTAANAC	504–535	(Cho et al., 1996; DasSarma & Fleischmann, 1995; Kumar et al., 2011; Lane, 1991; Liu et al., 2007; Makemson et al., 1997; Muyzer, De Waal & Uitterlinden, 1993; Nelson et al., 2014; Ovreås et al., 1997; Quince et al., 2011; Reysenbach & Pace, 1995; Ruff-Roberts, Kuenen & Ward, 1994; Walter et al., 2000; Wang & Qian, 2009; Wuyts et al., 2002)
5a	GTGTAGMGGTGAAATKCGTAGAT	682–704	(Klijn, Weerkamp & De Vos, 1991; Stults et al., 2001; Wang & Qian, 2009)
5b	CAAACRGGATTAGAWACCCNNGTAGTCCACGC	778–809	(Baker, Smith & Cowan, 2003; Barns et al., 1994; Brunk & Eis, 1998; Claesson et al., 2010; Colquhoun, 1997; Cruaud et al., 2014; DasSarma & Fleischmann, 1995; Engelbrektson et al., 2010; Flores et al., 2011; Lane, 1991; Liu et al., 2007; McBain et al., 2003; Nelson et al., 2014; Roesch et al., 2007; Sakai et al., 2004; Teske & Sorensen, 2007; Tremblay et al., 2015; Wang & Qian, 2009; Weisburg et al., 1991; Wilson, Blitchington & Greene, 1990)
6a	AAANTYAAANRAATWGRCGGGGRCCCGCACAAG	906–938	(Amann et al., 1992; Casamayor et al., 2002; Henckel, Friedrich & Conrad, 1999; Jurgens, Lindström & Saano, 1997; Lane et al., 1985; Liu et al., 2007; Mao et al., 2012; Nelson et al., 2014; Quince et al., 2011; Reysenbach & Pace, 1995; Rudi et al., 1997; Tremblay et al., 2015; Wang & Qian, 2009; Watanabe, Kodama & Harayama, 2001)
6b	ATGTGGTTTAATTCGA	948–963	(Iwamoto et al., 2000; Wang & Qian, 2009)
6c	CAACGCGARGAACCTTACC	966–984	(Jonasson, Olofsson & Monstein, 2002; Nübel et al., 1996; Sogin et al., 2006; Wang & Qian, 2009)
7a	AGGTGNTGCATGGYYGYCGTCAGCTCGTGYCGTGAG	1045–1080	(DasSarma & Fleischmann, 1995; Ferris, Muyzer & Ward, 1996; Huber et al., 2007; Jonasson, Olofsson & Monstein, 2002; Sogin et al., 2006; Wang & Qian, 2009; Youssef et al., 2009)
7b	TGTTGGGTTAAGTCCCRYAACGAGCGCAACCCT	1082–1114	(Cruaud et al., 2014; Ovreås et al., 1997; Reysenbach & Pace, 1995; Stackebrandt & Goodfellow, 1991; Wang & Qian, 2009; Wuyts et al., 2002)
8a	GGAAGGYGGGGAYGACG	1176–1192	(Bodenhausen, Horton & Bergelson, 2013; Wang & Qian, 2009)
8b	GGGCKACACACGYGCTAC	1219–1236	(DasSarma & Fleischmann, 1995; Youssef et al., 2009)
9	GCCTTGYACWCWCCGCCCGTC	1386–1406	(Kunin et al., 2010; Lane, 1991; Lane et al., 1985; Mao et al., 2012; Marchesi et al., 1998; Nübel et al., 1996; Reysenbach & Pace, 1995; Tremblay et al., 2015; Wuyts et al., 2002; Youssef et al., 2009; Yu & Morrison, 2004)
10	GGGTGAAGTCRTAACAAGGTANCC	1486–1509	(Hang et al., 2014; Isenbarger et al., 2008; Lin & Stahl, 1995; Oguntoyinbo, 2007; Reysenbach et al., 1992; Roesch et al., 2007; Weisburg et al., 1991; Wilson, Blitchington & Greene, 1990)

Except for primer contig 6b, the rest of primer contigs registered at least one degenerated base. Primer contig 3 was the most degenerated base-container sequence with eight degenerations, followed by primer contig 6a with seven and primer contig 4 with six and etcetera. The length of all primer contigs covered 388 nucleotides of the gene, which corresponded to 25% of the molecule size. Herein, 223 12-mers were generated; however this number increased to 2,886 after considering non-degenerated bases containing isomers (Table 2).

**Table 2:** Characteristics of primer contigs (Table 1) obtained after assembling all of the primers reported for each conserved region of the 16S rRNA gene. The number of possible 12-mers is size-dependent, while the number of isomers is related to the number of degenerated bases.

Name	Length	Degenerated bases	Number of 12-mers	Number of Iso 12-mers
1	20	2	9	36
2	20	3	9	61
3	44	8	33	488
4	32	6	21	970
5a	23	2	12	30
5b	32	4	21	306
6a	33	7	22	602
6b	16	0	5	5
6c	19	1	8	16
7a	36	5	25	167
7b	33	2	22	57
8a	17	2	6	22
8b	18	2	7	22
9	21	3	10	68
10	24	2	13	36
Total	388	49	223	2,886

### 12-mers search

When all iso12-mers, from each primer contig were searched in the sequences deposited in SILVA database release 123, highly variable coverages were revealed, which may explain in part the different results and PCR biases reported in these kinds of studies. Frequency analysis showed that extreme regions, 1 and 10, registered lower frequencies; for example, iso12-mers of these primer contigs were detected in less than 40% of the +513,000 sequences of SILVA (Table 3). These low frequencies detected at the extreme regions could be due to the interest focused on the central 16S rRNA regions. However, the most important factor is the absence of end segments (200–300 nucleotides) detected in several sequences of the database. This has been also reported in other studies (Wang et al., 2016; Yarza et al., 2014).

**Table 3:** 12-mers registering the highest frequency within each primer contig.

	12-mer		Frequency	
Primer contig	Number	Sequence	Number	Percent
01	8	ATYMTGGCTCAG	195,901	38.16%
02	1	SYGGCGNACGGG	405,570	79.01%
03	25	GGRNGGCNGCAG	500,253	97.46%
04	14	CVGCNGCYGCGG	496,412	96.71%
5a	5	GMGGTGAAATKC	382,156	74.45%
5b	10	TAGAWACCCNNG	493,348	96.11%
6a	10	RAATWGRCGGGG	501,792	97.76%
6b	2	GTGGTTTAATTC	389,530	75.89%
6c	6	GARGAACCTTAC	393,614	76.68%
7a	12	GYYGYCGTCAGC	499,976	97.40%
7b	20	CGAGCGCAACCC	489,290	95.32%
8a	3	AGGYGGGGAYGA	454,807	88.60%
8b	2	GCKACACACGYG	382,857	74.59%
09	5	GYACWCWCCGCC	388,911	75.77%
10	6	AGTCRTAACAAG	172,918	33.69%

**Figure 2:**
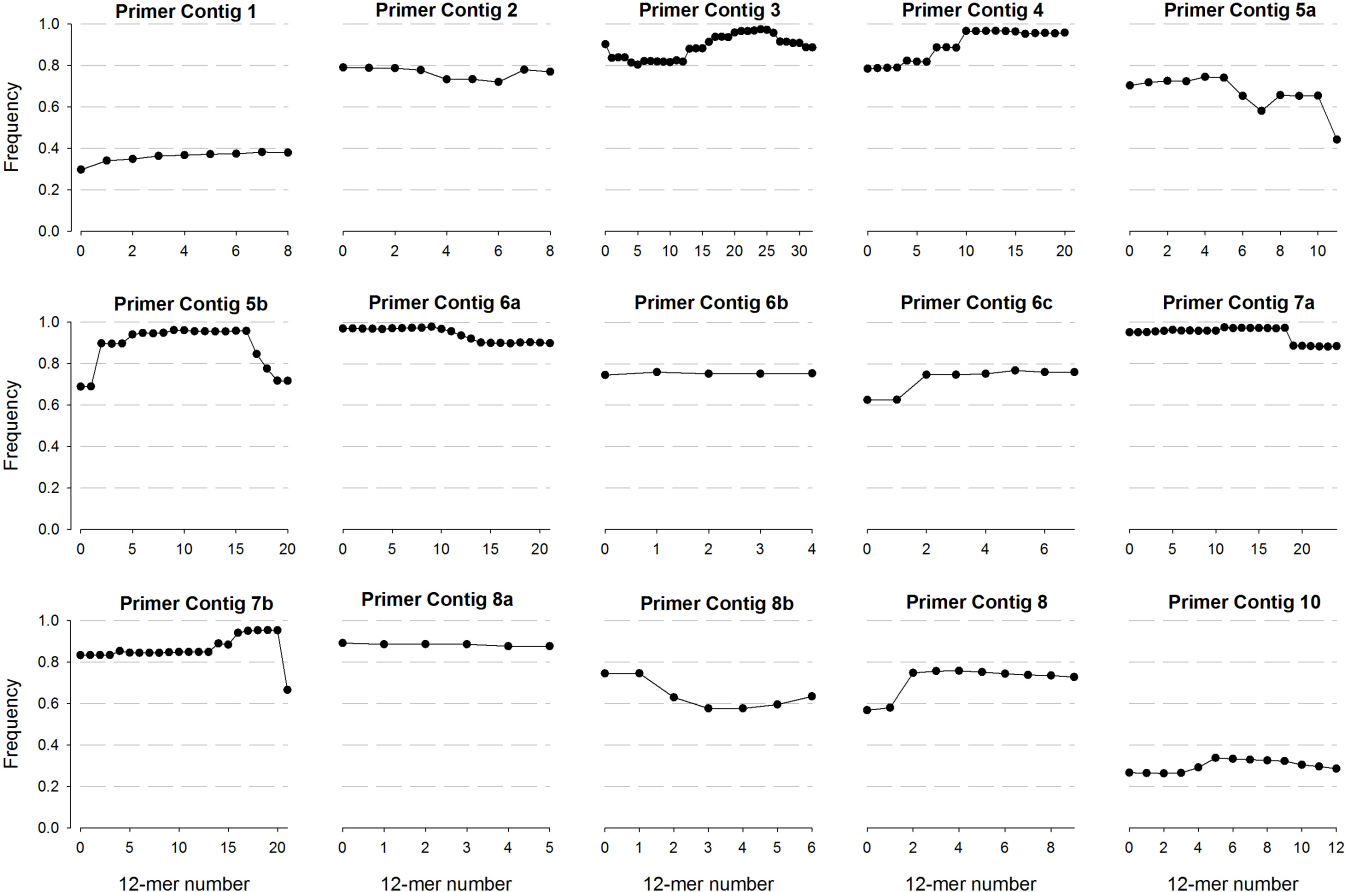
Frequency of 12-mers detected in contigs recovered from the different conserved regions of the 16S rRNA gene. More than one contig were recovered from a single conserved region.

Regarding more commonly used regions, the first 12-mers of primer contig 3 registered low frequencies (∼80%); however, this value raised up to 90% from position 16 and forward, reaching values of 97.5% (24th 12-kmer) (Fig. 2). A similar pattern was observed for primer contig 4 with a detection frequency of 78.5% and a progressive increase in forward direction, reaching values of 96.7% (13th 12-kmer). From the two primers contigs assembled in region 5, the 5a (positions 682 to 704) exhibited low frequencies for all 12-mers (60–80%); whereas higher frequencies were recorded for 5b (up to 96.1% in 9th 12-kmer) (Fig. 2). Regarding primer contigs from region 6 (6a, 6b and 6c), the highest frequency was detected for 12-mers of primer contig 6a, while none of 6b and 6c reached 80%. Two primer contigs were also detected for region 7 (7a and 7b), with the highest 12-kmer frequencies reported for 7a (95–97%) in the first segment of the molecule; however a frequency decrease was detected from 16th 12-kmer and forward; whereas the highest frequency detected for 7b was 95.3% (Fig. 2). Finally, primer contigs 2, 8a, 8b and 9 registered low frequency values, ranging from 60 to 85%.

Thus, considerable coverage variability was observed in conserved regions located at the extremes of the 16S rRNA gene (1 and 2, as well as 8, 9 and 10) where none of the 12-mers reached a frequency higher than 85%. These results could call into question the suitability of some of the primers that have been used for long time. In spite of these variabilities, 12-mers frequency analysis revealed that there are yet particular segments within each region with acceptable conservation degree to be considered for the study of prokaryotic diversity (Fig. 2). In this regard, 12-mers covering segments of regions 3, 4, 5b and 6a registered the highest frequencies, which could be useful information to design primers that are more suitable to profiling and comparing microbial communities; however, additional considerations have to be taken into account to design primers (Wang & Qian, 2009).

### Consensus Fragments Analysis

**Figure 3:**
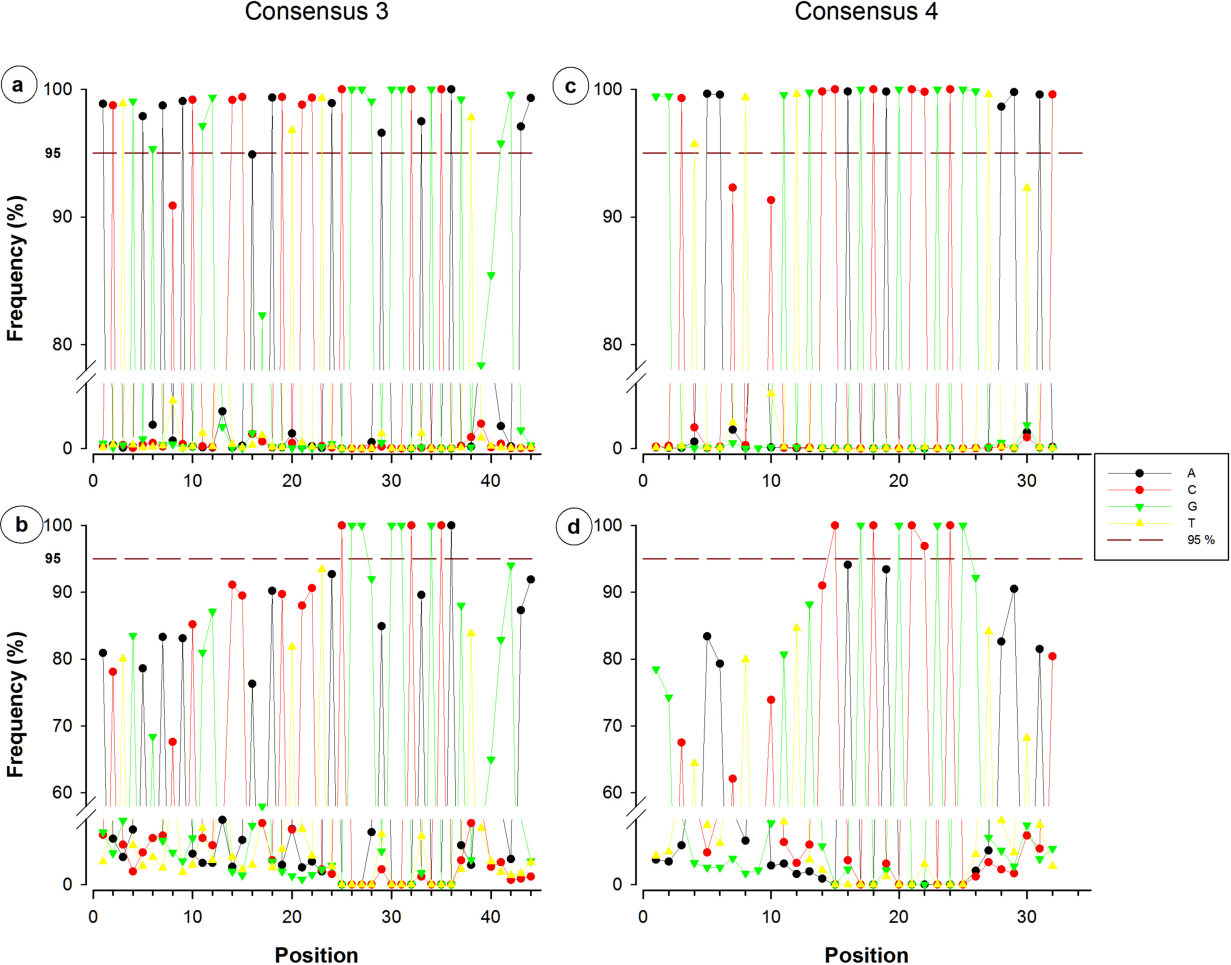
Single nucleotide frequencies for the consensus corresponding to primer contigs 3 and 4. Consensuses recovered from SILVA database are located on the upper side, whereas non-redundant sequences can be observed on the lower side. The red dotted line indicates the limit of 95%.

**Figure 4:**
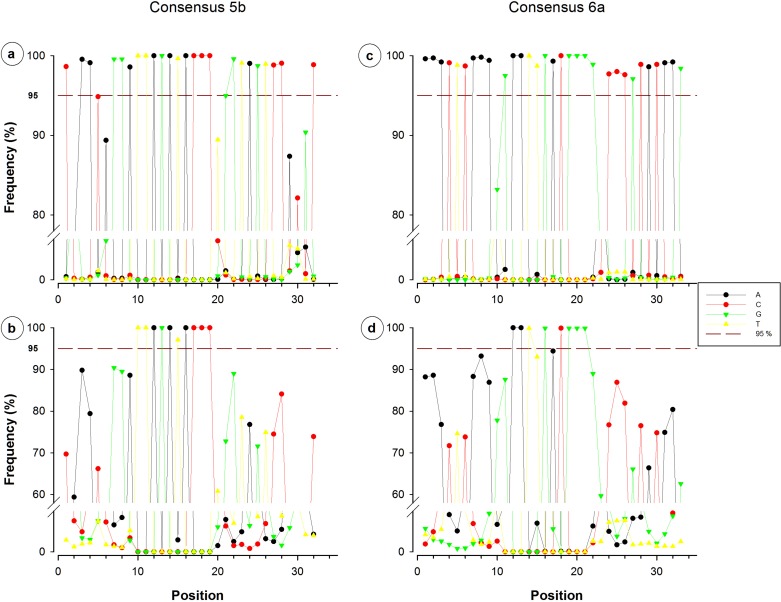
Single nucleotide frequencies for the consensus corresponding to primer contig 5b and 6a, recovered from all database SILVA (upper) and NR sequences (lower). Red dotted line indicates a limit of 95%.

In order to define the conservative sequences, each region corresponding to primer contig was recovered from more than 513,000 sequences in the SILVA database. The analysis was done for contigs 3, 4, 5b and 6a, because these regions constitute the most used target for primer design in 16S rRNA gene considered for the study of prokaryotic diversity. Around 500,000 fragments were recovered for each primer contig region and, after being manually aligned, the consensuses sized equal to the corresponding primer contig (32 to 44 nucleotides) detecting several degenerated bases. The frequency of each position for each aligned sequence set was determined (Figs. 3A, 3C, 4A and 4C). However, to avoid or reduce bias, subsequent analyzes were done with non-redundant sequences. The reduction in the number of sequences was significant; for example, the largest reduction (99.84%) was obtained with consensus 8a, where the 454,807 fragments obtained were pooled into 746 NR sequences. The lowest reduction in the number of sequence, from 500,253 to 11,586 (97.68%), was obtained by grouping consensus 3 fragments (Table 4). Each NR sequence represents a different number of fragments, and in some cases a small proportion is sufficient to represent the majority of the fragments. For consensus 3, more than 11,500 NR sequences were obtained, but only 498 are required to cover 95% of all retrieved fragments; in contrast, only 4 and 9 NR sequences are required to have a coverage of 95% of the consensuses 8a and 9, respectively (Table 4).

**Table 4:** Non-redundant (NR) sequences detected in consensuses corresponding to primer contigs. The last two columns indicate how many of NR sequences required to reach a coverage of 95% of corresponding fragment recovered from SILVA database.

Consensus	NR sequences		NR sequences needed to cover 95% of all fragments recovered	
	Number	Percent	Number	Percent
Consensus 2	1,844	0.45%	24	1.30%
Consensus 3	11,586	2.32%	498	4.30%
Consensus 4	4,323	0.87%	30	0.69%
Consensus 5b	6,694	1.36%	89	1.33%
Consensus 6a	5,301	1.06%	42	0.79%
Consensus 7a	4,312	0.86%	26	0.60%
Consensus 8a	746	0.16%	4	0.54%
Consensus 9	1,078	0.28%	9	0.83%

Nucleotide frequency analysis of consensuses revealed that small segments or single nucleotide positions were far to be constant within conserved regions. For instance, from the 44-nucleotide fragment conforming consensus 3, only five nucleotides registered a frequency below 95% (Fig. 3A), which would suggest a high conservation degree. However, when redundant sequences were eliminated and only NR sequences were considered for the analysis, 35 of the 44 nucleotides resulted to have a frequency below 95% (Fig. 3B). Only a 12-nucleotide segment containing nine nucleotides with frequency ≥95% was found to be the most conserved fraction of this region. A similar decrease of conserved nucleotides was observed in the other studied fragments when non-redundant sequences were considered, as shown in Figs. 3 and 4. Regarding consensus 4 sizing 32 nucleotides, an 11-nucleotide segment was detected to be the most conserved fragment with nine nucleotides registering frequencies above 95%; whereas fractions flanking this segment exhibited frequencies below 90% (Fig. 3D). A 10-nucleotide segment with ≥95% frequency was detected for consensus 5b constituted of 32 nucleotides (Fig. 4B); fractions flanking this segment registered extreme low frequency values, ranging from 10% to 90%. Similarly, a 10-nucleotide segment was found to be the most conserved fraction of consensus 6b composed by 33 nucleotides (Fig. 4D); from these 10 nucleotides, only eight resulted to be detected with a frequency above 95%, whereas nucleotides of fractions flanking the segment registered considerable variations (10–94%).

Despite all- and NR-sequences were analyzed in this trial, analyses based on NR sequences provided more realistic data, because all differences had same value; these kinds of approaches should be central to estimate how much conserved could be any region. Herein, primers complementing the conserved regions of the 16S rRNA gene of environmental prokaryotes are not necessarily complementary to all those that exist in the actual databases (Baker, Smith & Cowan, 2003). Degenerations have been used to include all these new sequences; however this replacement may difficult the design of adequate primers and reduces the confidence of conserved regions. Furthermore, the low iso 12-mers frequencies in SILVA sequences could be associated to a greater proportion of degenerations than those actually reported, or inclusively to the potential presence of sequencing errors; for example, any insertion or deletion of a single nucleotide cause shifting of the entire sequence and reports biased diversity.

The single nucleotide-frequency analysis provided additional information that revealed the most conserved nucleotide positions within each consensus. These results revealed that most of the positions of the conservative regions were not as conserved as expected. Herein, the sum of the consensuses covered 25% of the molecule (388 nucleotides); however, only 75 nucleotides showed frequencies of at least 95%, representing 5% of the molecule size. Such information revealed that a very small fraction 16S rRNA gene is truly conserved (≥95%); therefore, primer design must necessarily be anchored to these very short, but highly conserved segments. Furthermore, these short segments corresponded to the 12-mers that registered the highest frequencies. Table 5 shows nucleotide pattern of each of the studied regions (2, 3, 4, 5b, 6a, 7a, 8a and 9) considering the sequences of SILVA database, the sets of NR sequences, as well as primer contig for comparison.

**Table 5:** The primer contig and the corresponding consensuses from both all and non-redundant sequences are aligned. Conserved nucleotides in the consensus of all sequences are in blue, while those preserved in non-redundant sequences are in red.

2	Primer contig	**ASYGGCGNACGGGTGAGTAA**
	All sequences	**ASYGGCGVACGGGTGMGTAA**
	NR sequences	**ASYGGCGVACGGBNNNNNNN**
3	Primer contig	**ACTGAGAYACGGYCCARACTCCTACGGRNGGCNGCAGTRRGGAA**
	All sequences	**ACTGAGAYACGGHCCRRACTCCTACGGGAGGCAGCAGTVRGGAA**
	NR sequences	**VHBDVVVHVVBBNYMVNVHHYHYRCGGRDGGCWGCAVBNDVRRR**
4	Primer contig	**GGCTAACTHCGTGNCVGCNGCYGCGGTAANAC**
	All sequences	**GGCTAAYTHYGTGCCAGCAGCCGCGGTAAKAC**
	NR sequences	**BBNHHHHHHBBKBSCMGCMGCCGCGKDDWNHV**
5b	Primer contig	**CAAACRGGATTAGAWACCCNNGTAGTCCACGC**
	All sequences	**CRAAYRGGATTAGATACCCYGGTAGTCCWHRC**
	NR sequences	**VVVVNVRRHTTAGATACCCBNKDDDBBHNNVV**
6a	Primer contig	**AAANTYAAANRAATWGRCGGGGRCCCGCACAAG**
	All sequences	**AAACTCAAAKGAATTGACGGGGRCCCGCACAAG**
	NR sequences	**DHHHHHMRRDRAATWGRCGGGRVNBBVVVMVVV**
7a	Primer contig	**AGGTGNTGCATGGYYGYCGTCAGCTCGTGYCGTGAG**
	All sequences	**AGGTGSTGCATGGYTGTCGTCAGCTCGTGTCGTGAG**
	NR sequences	**VDDBBNBSMWYGGYYGTCGTCAGYYBBBDBBBBDDR**
8a	Primer contig	**GGAAGGYGGGGAYGACG**
	All sequences	**GGAAGGTGGGGATGACG**
	NR sequences	**GGAAGGYGGGGANNNNN**
9	Primer contig	**GCCTTGYACWCWCCGCCCGTC**
	All sequences	**GYCTTGYACWCACCGCCCGTC**
	NR sequences	**NNNBTGYACWCWCCGCHNNNN**

These analyses could be also used as a methodological pathway focused to particular environments, and selecting only the inhabitants reported in specific databases, like the marine waters with their particular microorganisms, or exclusive microbial pathogens that require more specific discrimination. It is important to consider that only fractions of the entire bacteria species could be detected in different environments, and that from such diversity some kinds of bacteria would have a greater representation than others; for this reason the use of NR sequences improves coverage of bacteria thriving into random environments. Finally, it could be concluded that conserved regions of the 16S rRNA gene exhibit considerable variations; however, it was demonstrated that it is possible to achieve more reliable primers designs.

##  Supplemental Information

10.7717/peerj.3036/supp-1 Supplemental Information 1 List of iso 12-mers, frequencies for 12-mers and nucleotides List of all the generated iso 12mers for each primer set, as well as the list of frequencies for 12mers and the frequency of nucleotide distribution at each consensus position.

## Additional Information and Declarations

### Data Availability

The following information was supplied regarding data availability: The raw data has been supplied as a Supplementary File.
